# ﻿A systematic review of the genus *Bolbelasmus* Boucomont (Coleoptera, Geotrupidae, Bolboceratinae) from Indochina and surrounding areas

**DOI:** 10.3897/zookeys.1191.114021

**Published:** 2024-02-14

**Authors:** Chun-Lin Li, Chuan-Chan Wang

**Affiliations:** 1 The Experimental Forest, National Taiwan University, Chushan, Nantou County 557, Taiwan National Taiwan University Nantou County Taiwan; 2 Department of Life Science, Fu Jen Catholic University, Hsinchuang, New Taipei City 24205, Taiwan Fu Jen Catholic University New Taipei City Taiwan

**Keywords:** Checklist, earth-boring beetles, key, natural history, new species, taxonomy

## Abstract

Indochinese species of the genus *Bolbelasmus* (Coleoptera, Geotrupidae, Bolboceratinae) are reviewed. Three new species, *Bolbelasmuschifengi* Wang & Li, **sp. nov.**, *Bolbelasmusconcavisuturalis* Li & Wang, **sp. nov.** and *Bolbelasmusyutangi* Li & Wang, **sp. nov.**, are described and illustrated. An annotated checklist and modified key to species of the genus are provided. Information for each species in the checklist includes literature review, synonymy, distribution and type locality.

## ﻿Introduction

*Bolbelasmus* Boucomont, 1910 (Coleoptera: Geotrupidae: Bolboceratinae: Bolboceratini) is one of the largest bolboceratine genera, currently consisting of 29 species and two subspecies (Schoolmeesters 2023) (including subgenus Kolbeus). Among bolboceratine genera, *Bolbelasmus* has the widest distribution, occurring throughout the Holarctic and Oriental regions (Li et al. 2008; Hillert et al. 2016). Krikken (1977) published the most recent review dealing with eastern and southeastern Asian species and provided a complete checklist and summary of generic affinities and notes of species from the Middle East and North Africa. In that paper, two new species were described including one, *Bolbelasmusmeridionalis* Krikken, 1977, from Southeast Asia. Additional distribution data were provided for *B.coreanus* (Kolbe, 1886) in southwestern China, Thailand and India. The Indochinese *Bolbelasmus* species have received little attention since Krikken’s (1977) work. Zinchenko (2016) described a new species, *B.korshunovi* Zinchenko, from northern Thailand, the only species described during the past four decades. Currently, ten species are recorded in eastern and southeastern Asia, including the three new species described here based on specimens recently obtained from the Indochinese Peninsula and neighboring areas, including the first record from Myanmar. These collections constitute the basis for further detailed knowledge of the Indochinese bolboceratine fauna.

The natural histories of eastern and southeastern Asian *Bolbelasmus* species are poorly documented. Tsukamoto et al. (2017) reported that two of the Japanese species, *B.nativusishigakiensis* Masumoto and *B.shibatai* Masumoto inhabit densely forested montane areas, where adults are nocturnal and fly about 30 cm over the forest floor from late dusk to midnight. Adults of both species can be sporadically attracted to light, as observed with *B.coreanus* (Kolbe) in Taiwan (Li et al. 2008). *Bolbelasmus* specimens can occasionally be collected using baited traps (e.g., Kawai et al. 2005; Li et al. 2008).

In this paper, we review the species of *Bolbelasmus* occurring in the Indochinese Peninsula and neighboring areas, with descriptions of three new species. We also provide an annotated checklist, distributional data for known species in eastern and southeastern Asia, and a modified key.

## ﻿Material and methods

The type specimens of the two new species, *B.chifengi* and *B.yutangi*, were collected by flight interception traps (FIT). The depository of the type specimens is in the following institutions: National Museum of Nature and Science (NSMT, Tsukuba, Japan); Zoological Museum, University of Copenhagen (ZMUC, Copenhagen, Denmark); Taiwan Agriculture Research Institute (TARI, Taichung, Taiwan); and the private collection of Chun-lin Li (CCLI, Nantou, Taiwan). Habitus images of *Bolbelasmus* specimens were taken using a Canon 7D digital camera with a Canon EF 100 mm macro lens and a Canon Macro Twin Lite MT-24EX Flash. Detailed images of specimens, body parts and male genitalia were captured using a Leica M205C stereo microscope equipped with a Leica MC190HD microscope camera or by a Hitachi TM3030 Plus tabletop scanning electron microscope. Color images were processed using Helicon Focus v.8.2.0 to increase depth of field, and all images were edited in Adobe Photoshop v.24.0.0 (background removed, images integrated, numbered and scale bars added). Measurements, treatments, and preservation of specimens and genitalia are based on methods described by Li et al. (2008).

## ﻿Taxonomy

### ﻿ Bolbelasmus

Taxon classificationAnimaliaColeopteraGeotrupidae

Boucomont, 1910

B508A3A2-4FAB-59BF-84BC-FEC207B96689

#### Diagnosis.

Species of *Bolbelasmus* are small to medium-sized (5.6–15.2 mm in body length) and can be recognized by the glossy, unicolored, reddish-brown to black dorsal surface; presence of a conical frontal tubercle with a rounded or bifurcated tip in males; transverse frontal carina present in females; eyes protruding, divided by a canthus anteriorly, canthus with anterior margin smooth; antennal club with first segment glabrous on inner side; pronotum usually quadrituberculate in major males (vestigial or reduced to bituberculate in minor males), females with transverse carina only; first elytral stria terminated by scutellum; parameres usually weakly sclerotized.

### ﻿Key to eastern and southeastern Asian *Bolbelasmus* species based on males

(excluding *Bolbelasmusorientalis*)

**Table:** 

1	Frontal tubercle located at center of frons (Fig. 15)	**2**
–	Frontal tubercle located in junction of clypeofrontal suture (Figs 11–14, 16)	**4**
2	Sutural intervals of elytra more convex than other intervals (Figs 25, 26)	**3**
–	Sutural intervals (Fig. 23, 24, 27, 28) of elytra equally convex as other intervals; pronotal median tubercles well developed, primary punctures moderately distributed throughout disc (Fig. 21) except for a small area near posterior margin impunctate; posterior margin punctate; parameres small, narrowed apically, curved in lateral view	***Bolbelasmusyutangi* sp. nov.**
3	Pronotal median tubercles weakly developed, center of disc typically impunctate, primary punctures moderately distributed at sides of center, posterior margin sparsely punctate at center; parameres small, narrowed apically, flat in lateral view	***Bolbelasmusnativus* Krikken, 1979**
–	Pronotal median tubercles well developed, disc sparsely punctate, primary punctures sparsely distributed at sides of center (Fig. 20), posterior margin impunctate at center; parameres (Figs 35, 36, 42) moderate in size, trapezoidal, bases swollen in lateral view	***Bolbelasmuskorshunovi* Zinchenko, 2016**
4	Elytral sutural intervals (Fig. 25) distinctly convex	**5**
–	Elytral sutural intervals concave (Fig. 23), flat or partially convex (Figs 24, 27, 28)	**6**
5	Lateral margins of pronotum widely explanate; parameres with tips angulate at anterolateral angles	***Bolbelasmusmeridionalis* Krikken, 1979**
–	Lateral margins of pronotum narrowly explanate; parameres with tips narrowed anteriorly	***Bolbelasmusminutus* Li & Masumoto, 2008**
6	Elytral sutural intervals completely concave; parameres (Figs 29, 30, 39) small, anteriorly 1/2 curved ventrally in lateral view	***Bolbelasmusconcavisuturalis* sp. nov.**
–	Elytral sutural intervals flat or partially, moderately convex	**7**
7	Elytral sutural intervals partially, moderately convex; parameres with bases contracted in dorsal view, length longer than one-half of basal piece	**8**
–	Elytral sutural intervals flat; parameres straight, small, length shorter than one-half of basal piece	***Bolbelasmuskrikkeni* Nikolajev, 1979**
8	Pronotal disc sparsely punctate	**9**
–	Pronotal disc with many primary punctures (Fig. 18); parameres (Figs 31, 32) large with tips acute and vertically curved inward	***Bolbelasmuschifengi* sp. nov.**
9	Pronotal disc with primary punctures finer (Fig. 22); elytral intervals (Fig. 28) slightly convex; parameres with inner margins straight and separate, moderately evenly sclerotized	***Bolbelasmuscoreanus* (Kolbe, 1886)**
–	Pronotal disc with primary punctures coarser; elytral intervals convex; parameres with inner margins broadened basally and overlapping, distinctly sclerotized, partly with median lobe	***Bolbelasmusshibatai* Masumoto, 1984**

### ﻿Checklist of species of *Bolbelasmus* Boucomont from eastern and southeastern Asia

### ﻿ Bolbelasmus chifengi

Taxon classificationAnimaliaColeopteraGeotrupidae

Wang & Li sp. nov.

13FA9162-948C-5A5A-92FF-18A5591DFCB1

https://zoobank.org/624857C3-4224-46EF-80FA-FDD3E4B1F169

Figs 3 , 4 , 12 , 18 , 24 , 31 , 32 , 40 , 46 , 47

#### Type materials.

***Holotype* male.** “China: Yunnan, Bangdashan (邦達山), 16.IX.2015. leg. Y.-T. Wang.” (glued on label, TARI), Taichung, Taiwan. ***Paratypes*.** 3♂♂, 1♀(TARI). same collecting data as the holotype. 1♀ (TARI). “China: Yunnan, Wudian (武甸), 17.IX.2014. leg. Y.-T. Wang”. 1♂, 2♀♀(TARI). “China: Yunnan, CCCC, Nabang (那邦), 21.VI.2017. leg. Y.-T. Wang”. 1♀ (CCLI). “China: Yunnan, Banggunjianshan (邦棍尖山), 19.IX.2015. leg. Y.-T. Wang”. 1♂ (CCLI). “China: Yunnan, Bangdashan (邦達山), 01.IX.2015. leg. Y.-T. Wang”. 2♂♂, 1♀(CCLI). “China: Yunnan, Ruili (瑞麗), 01.IX.2014. leg. Y.-T. Wang”.

#### Description.

**Holotype male** (Figs 3, 4). Body length 9.7 mm; width across humeri 6.0 mm. Dorsum moderately shiny. Head, pronotum and scutellum dark brown with elytra reddish brown. ***Head*** (Fig. 12): labrum with anterior margin crenulate, disc transversally rugose. Clypeus trapezoidal, surface densely rugopunctate; protrusion at basal angle moderately developed. Clypeofrontal suture well defined, distinctly curved in front of frontal tubercle. Frons with surface sparsely punctate, punctures fine, frontal tubercle vertically located in junction of suture, right-triangle in shape in lateral view. Eye prominent, canthus simple, not exceeding eye. ***Thorax***: pronotum (Fig. 18) quadrituberculate, tubercles placed in a line, lateral tubercle greatly reduced in size; anterior face of median tubercles almost perpendicular to plane of pronotum; primary punctures coarse, moderately distributed on disc and intermixed with impunctate area, punctures between lateral margins of pronotum and fovea bigger and denser, posterior area between elytral humeri and suture impunctate except for four coarse punctures in front of scutellum, secondary punctures tiny, evenly scattered throughout surface of pronotum; frontal and lateral margins beaded, posterior margin beaded only in front of elytral humeri. Scutellum elongate, secondary punctures sparse throughout surface with a coarse puncture at center. ***Elytron*** (Fig. 24): elytral striae shallow, punctures mostly spaced 2–3 times diameters of punctures. Intervals slightly convex including sutural one, surface with scattered secondary punctures. Male genitalia (Figs 31, 32, 40).

**Female** (Figs 46, 47). Body length 7.7–10.0 mm; width across humeri 5.4–6.5 mm. Similar to male with minor differences in the form of strongly wrinkled surface of clypeus, transverse frontal carina trilobed, central lobe more prominent than lateral lobes, punctures on frons and vertex rugose, transverse pronotal carina feebly bilobed with lobes broadly developed, punctures on pronotal disc coarser and denser than males.

**Variation in male.** Dorsum brown, smaller body size, 6.6 mm in length and 5.2 mm in width across humeri, frontal tubercle less developed and not in junction of clypeofrontal suture, pronotal tubercles feebly convex, and number of coarse punctures arranged at pronotal posterior margin variable.

#### Diagnosis.

*Bolbelasmuschifengi* is morphologically similar to *B.concavisuturalis*, but can be distinguished from the latter by the coarser and denser primary punctures on the pronotal disc (finer and scattered in *B.concavisuturalis*), elytral intervals evenly convex (elytral intervals flat with sutural interval concave in *B.concavisuturalis*) and by the longer parameres (shorter in *B.concavisuturalis*).

#### Distribution.

Southern Yunnan, China (Fig. 52).

#### Etymology.

*Bolbelasmuschifengi* sp. nov. is named after Dr Chi-feng Lee, the curator of the Department of Applied Zoology, Taiwan Agriculture Research Institute, Taichung, Taiwan, who kindly provided materials used in this study.

### ﻿ Bolbelasmus concavisuturalis

Taxon classificationAnimaliaColeopteraGeotrupidae

Li & Wang sp. nov.

04B3E5DB-EF5D-5AC6-8E22-05860EBA22D6

https://zoobank.org/B3BDDA3C-0CE3-4ACF-9470-E2750D968B93

Figs 1 , 2 , 11 , 17 , 23 , 29 , 30 , 39 , 44 , 45

#### Type materials.

***Holotype* male.** “Mon-Angget, near Chiangmai, North Thailand, 31-V-1990, K. Masumoto leg.” (glued on label, NSMT). ***Paratypes*.** 1♂ (ZMUC) “Northern Thailand, Doi Sutep, 21.6.1958, B. Degerbøl leg., Pr. 548 (1.7.59)”. 1♀ (NSMT) “Doi Suthep, Chiang Mai, Thailand, 15-VIII-1989, Y. MANIT leg.”

#### Description.

**Figures 1–6.**
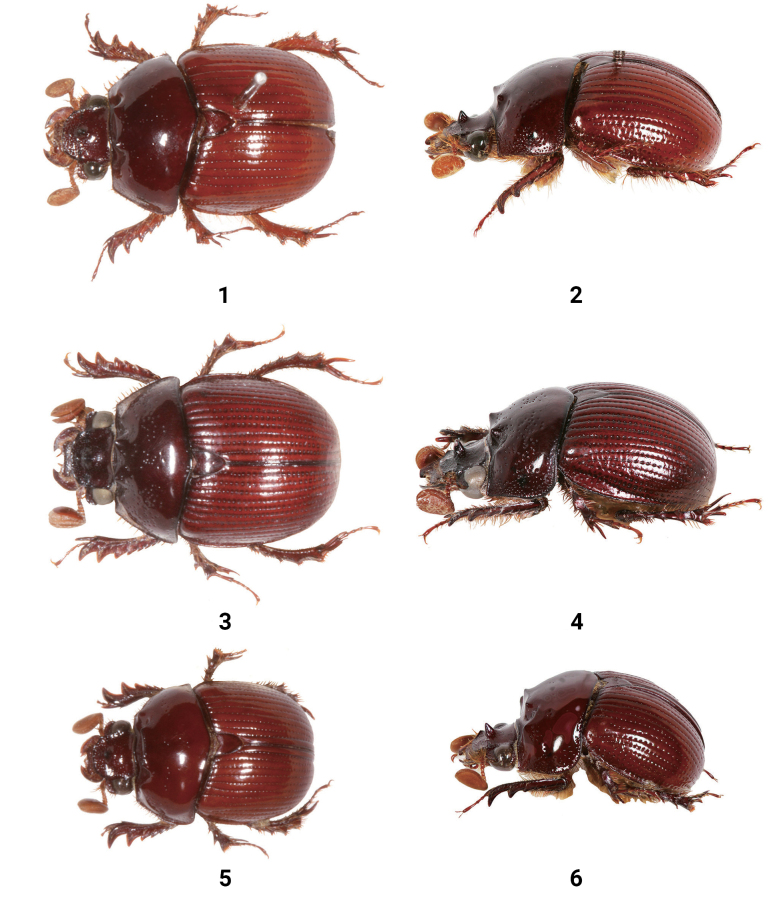
Dorsal and lateral views of male *Bolbelasmus* spp. **1, 2***B.concavisuturalis* sp. nov.sp. nov., holotype **3, 4***B.chifengi* sp. nov., holotype **5, 6***B.meridionalis*.

**Holotype male** (Figs 1, 2). Body length 10.2 mm; width across humeri 6.3 mm. Dorsum distinctly shiny. Head, pronotum and scutellum reddish brown with elytra brown in color. ***Head*** (Fig. 11): labrum with anterior margin crenulate, disc transversally rugose. Clypeus trapezoidal, surface densely rugopunctate; protrusion at basal angle reduced. Clypeofrontal suture well defined, distinctly curved in front of frontal tubercle. Frons with surface sparsely punctate, punctures fine, frontal tubercle vertically located in junction of suture, triangular in lateral view. Eye prominent, canthus simple, not exceeding eye. ***Thorax***: pronotum (Fig. 17) quadrituberculate, tubercles situated in a line, lateral tubercle smaller; anterior face of median tubercles almost perpendicular to surface of pronotum; primary punctures weakly defined, sparse on disc except between lateral margin of pronotum and fovea, these coarser and denser, line in front of scutellum with a coarse puncture, secondary punctures tiny, evenly scattered on surface of pronotum; frontal and lateral margins beaded, posterior margin beaded only anterior to humeri of elytra. Scutellum elongate, secondary punctures sparsely distributed. ***Elytron*** (Fig. 23): elytral striae shallowly impressed, punctures mostly spaced 2–3 times diameters of punctures. Intervals flat, with sutural interval weakly concave, surface scattered with secondary punctures. Male genitalia (Figs 29, 30, 39).

**Figures 7–10.**
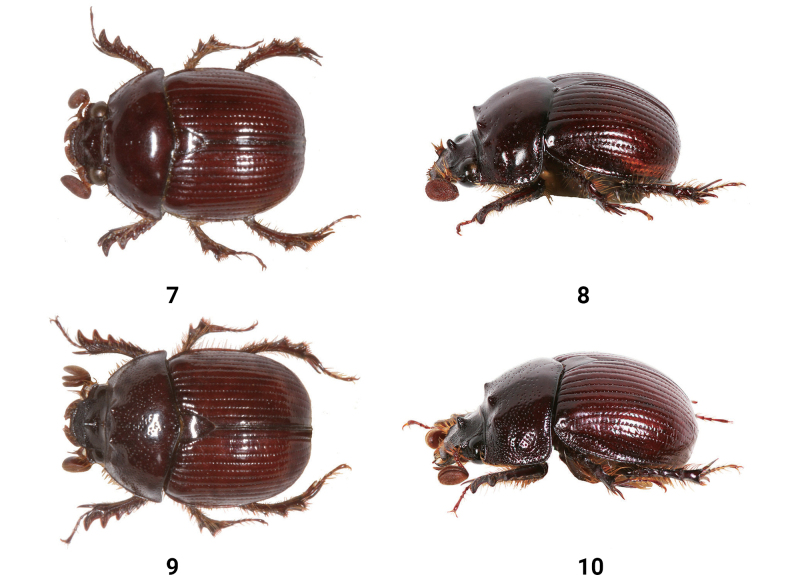
Dorsal and lateral views of male *Bolbelasmus* spp. **7, 8***B.korshunovi***9, 10***B.yutangi* sp. nov., holotype.

**Figures 11–16.**
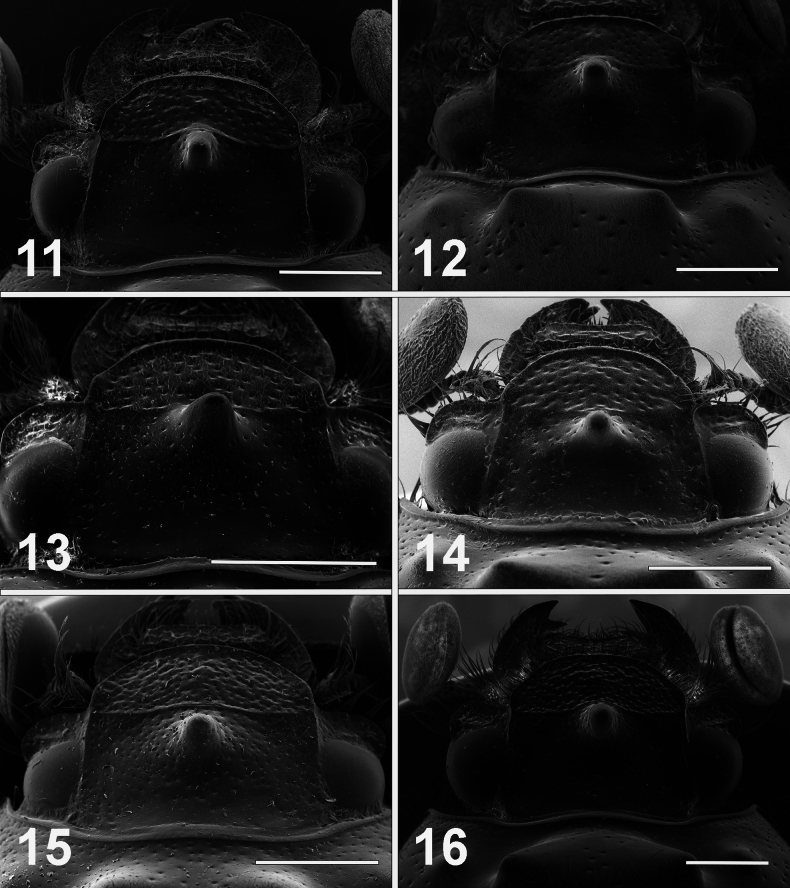
Scanning electron micrographs of heads of male *Bolbelasmus* spp. **11***B.concavisuturalis* sp. nov. **12***B.chifengi* sp. nov. **13***B.meridionalis***14***B.korshunovi***15***B.yutangi* sp. nov. **16***B.coreanus*. Scale bar: 1 mm.

**Female paratype** (Figs 44, 45). Body length 9.8 mm; width across humeri 5.8 mm. Similar to male with minor differences in the form of strongly wrinkled surface of clypeus, transverse frontal carina trilobed, central lobe more prominent than lateral lobes, punctures on frons and vertex rugose, transverse pronotal carina feebly bilobed, lobes broad, punctures on pronotal disc coarser and denser than those of males.

**Male paratype.** The single male paratype is smaller in body size, 9.4 mm in length and 5.1 mm in width across humeri, frontal tubercle less developed and with three coarse punctures along pronotal posterior margin in front of scutellum.

#### Diagnosis.

**Figures 17–22.**
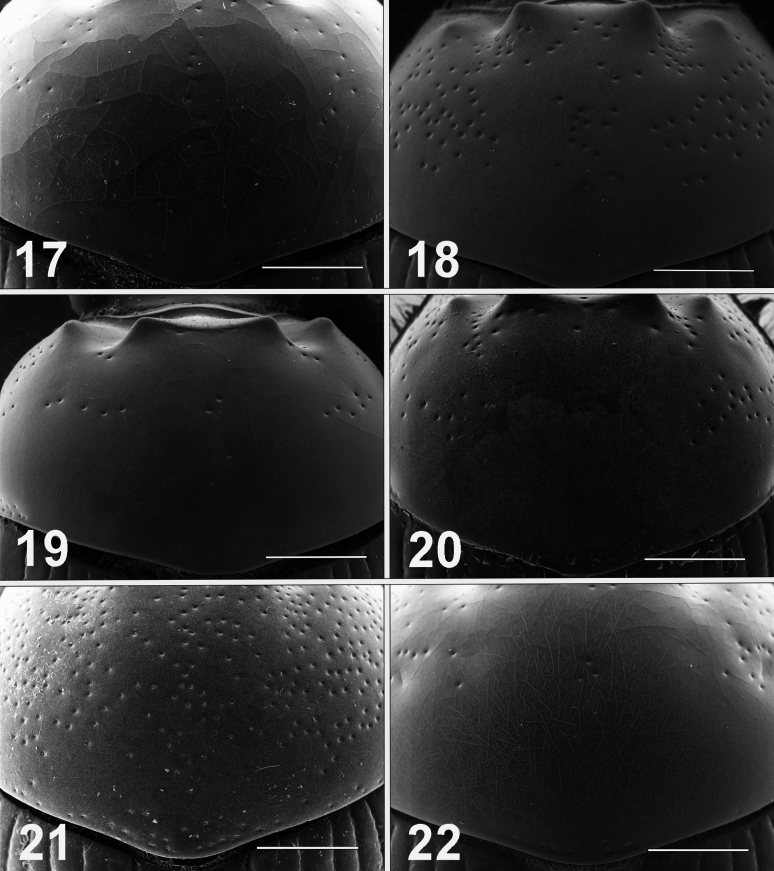
Scanning electron micrographs of pronota of male *Bolbelasmus* spp. **17***B.concavisuturalis* sp. nov. **18***B.chifengi* sp. nov. **19***B.meridionalis***20***B.korshunovi***21***B.yutangi* sp. nov. **22***B.coreanus*. Scale bar: 1 mm.

**Figures 23–28.**
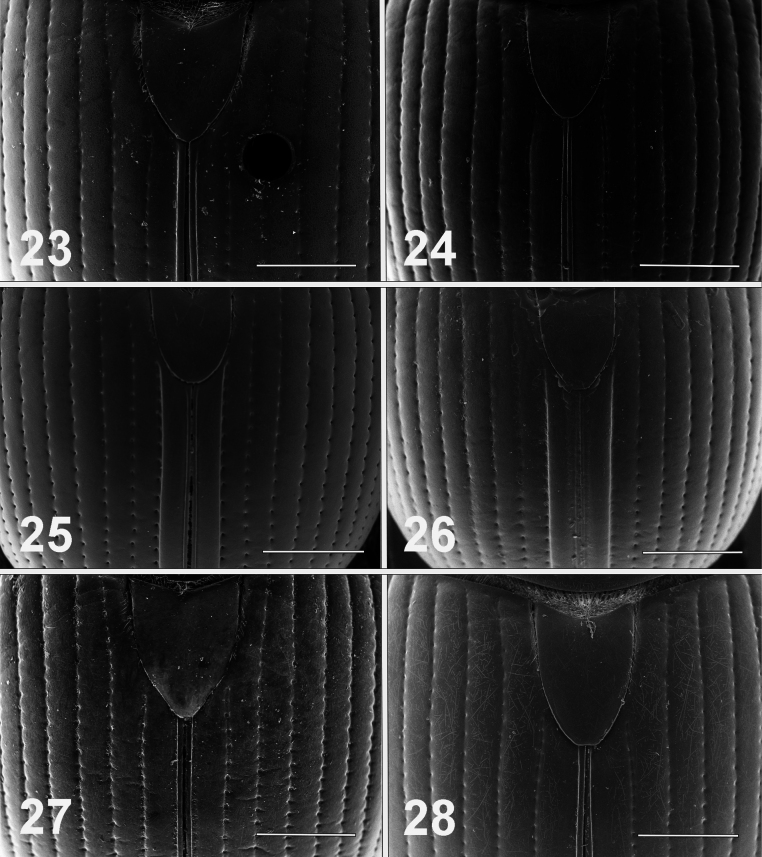
Scanning electron micrographs of elytra of male *Bolbelasmus* spp. **23***B.concavisuturalis* sp. nov. **24***B.chifengi* sp. nov. **25***B.meridionalis***26***B.korshunovi***27***B.yutangi* sp. nov. **28***B.coreanus*. Scale bar: 1 mm.

*Bolbelasmusconcavisuturalis* sp. nov. is morphologically similar to *B.coreanus*, but can be distinguished from the latter by having denser punctures along the midline of the pronotum (Fig. 17) (sparser punctures in *B.coreanus* (Fig. 22)), punctures in elytral striae moderately developed (Fig. 23) (punctures weakly developed in *B.coreanus* (Fig. 28)) and ventrally curved parameres (straight in *B.coreanus*).

#### Distribution.

Northern Thailand (Fig. 52).

#### Etymology.

*Concavi*- (L.) = concave, -*suturalis* (L.) = suture. In reference to the concave sutural intervals of the elytra.

### ﻿ Bolbelasmus coreanus

Taxon classificationAnimaliaColeopteraGeotrupidae

(Kolbe, 1886)

6A70B116-BB56-5A2B-A23B-6323990FDA36

Bolboceras coreanus Kolbe, 1886: 188. Original combination (type locality: Seoul, Korea, female type in the Museum für Naturkunde, Berlin, Germany).
Bolbelasmus coreanus (Kolbe, 1886): Cartwright 1953: 97 (generic combination); Krikken 1977: 288 (notes; diagnosis; illustration); Kim 2000: 45 (diagnosis; collecting records); Li et al. 2008: 480 (redescription, illustrations, collecting records, distribution, remarks); Král, Löbl and Nikolajev 2006: 83 (catalog, subgenus Kolbeus); Nikolajev, Král and Bezdӗk 2016: 33 (catalog, subgenus Kolbeus).
Kolbeus coreanus (Kolbe, 1886): Boucomont 1911: 335 (generic combination); Boucomont 1912: 17 (catalog); Boucomont and Gillet 1921: 72 (record to Taiwan; diagnosis); Miwa 1930: 164 (catalog); Miwa 1931: 276 (catalog); Miwa and Chûjô 1939: 30 (catalog); Paulian 1945: 42 (diagnosis; figures; distribution).
Bolbelasmus kurosawai Masumoto, 1984: 76; Li et al. 2008: 481 (junior synonym).
Bolboceras conicifrons Fairmaire, 1896: 82; Boucomont and Gillet 1921: 72 (junior synonym).

#### Distribution.

Korean Peninsula; China (Anhui, Zhejiang, Kweichow, Szechuan, Yunnan); Taiwan.

#### Remarks.

The detailed distribution of *B.coreanus* in China requires further investigation, particularly those from southwestern areas. Based on a large number of *Bolbelasmus* specimens recently collected from Yunnan and neighboring areas, we found no representatives of *B.coreanus* among them. Therefore, we reserve a decision about whether *B.coreanus* occurs in Yunnan, Thailand and India, as recorded by Krikken (1977). Voucher specimens from the areas mentioned above are required.

### ﻿ Bolbelasmus korshunovi

Taxon classificationAnimaliaColeopteraGeotrupidae

Zinchenko, 2016

8409E5B6-8AF9-5ABB-8545-DE166EA5384F

Figs 7 , 8 , 14 , 20 , 26 , 35 , 36 , 42

Bolbelasmus korshunovi Zinchenko, 2016: 328. Original combination (type locality: Nong Bun Nak, Nakhon Prov., Thailand).

#### Material examined.

(5♂♂). 2♂♂ (ZMUC). Thailand: Loei Province, Phu Luang Wildlife Sanctuary, 8.–14.x.1984, 700–900 m, Karsholt, Lomboldt & Nielsen leg., Pral Siaw, 1923-9-33, Paūl Fogh/ Coll. Roseberg. 3♂♂ (NSMT). Sansai, Chiang Mai, Thailand, 17. VI. 1993.

#### Diagnosis.

Body length, males, 6.8–9.0 mm, greatest width at pronotal base 4.4–5.7 mm; females, 7.1–8.7 mm in length, 4.3–5.6 mm in width (Zinchenko 2016). *Bolbelasmuskorshunovi* is distinguished from the other Oriental *Bolbelasmus* species by elytral sutural intervals that are moderately convex, primary punctures sparsely distributed either side of the center of the pronotum, and shapes of the parameres.

#### Notes.

Thirteen type specimens were designated in the original description of the species (Zinchenko 2016), 12 of them collected from June to August, and the holotype during November. Accordingly, the temporal activity of adults is likely at least half the year during both rainy and dry seasons. This is identical to the sympatric species, *B.meridionalis*.

#### Distribution.

Northern Thailand.

#### Remarks.

*Bolbelasmuskorshunovi* inhabits plains to low-elevational montane areas and occurs sympatrically with *B.meridionalis* in northern Thailand.

### ﻿ Bolbelasmus krikkeni

Taxon classificationAnimaliaColeopteraGeotrupidae

Nikolajev, 1979

6B60480D-F665-50FF-AC3D-424931B0F1D0

Bolbelasmus krikkeni Nikolajev, 1979: 225. Original combination (type locality: Gopaldhara, Sikkim, India); Král, Löbl and Nikolajev 2006: 83 (catalog, in subgenus Kolbeus); Nikolajev, Král and Bezdӗk 2016: 33 (catalog, subgenus Kolbeus).

#### Distribution.

Northern India.

#### Remarks.

Based on the collecting data from the monotypic specimen, *B.krikkeni* occurs in mid-elevation forests above 1000 m and is unique compared to its congeners that usually inhabit plains to low-elevation montane areas in the region. No additional specimens have been recorded since the publication of the original description.

### ﻿ Bolbelasmus meridionalis

Taxon classificationAnimaliaColeopteraGeotrupidae

Krikken, 1977

E0B0EB6A-0CC3-5F4C-A382-A1C954C295D7

Figs 5 , 6 , 13 , 19 , 25 , 33 , 34 , 41 , 48 , 49

Bolbelasmus meridionalis Krikken, 1977: 285. Original combination (type locality: Java, Indonesia); Král, Löbl and Nikolajev 2006: 83 (catalog, subgenus Kolbeus); Nikolajev, Král and Bezdӗk 2016: 33 (catalog, subgenus Kolbeus).

#### Material examined.

(11♂♂, 8♀♀). 1♂ (NSMT). Thailand: Sansai, Chiang Mai, 17. VI. 1993 (1 male at NSMT). 9♂♂, 7♀♀ (NSMT); same locality, 12-V-1996. 1♀ (NSMT); near Chiang Mai, N Thailand, VII-1996, native collector. 1♂ (NSMT). Taiwan: Formosa, Heito, 10-VII-1941, H. Kondo/Sizumu Nomura Bequest, 1981.

#### Diagnosis.

Body length, males, 6.1–8.2 mm, greatest width at pronotal base, 3.7–5.0 mm; females, 5.6–8.2 mm in length, 3.3–5.2 mm in width. Both *B.meridionalis* and *B.minutus* constitute a distinctive group among southeastern Asian congeners based on sharing the distinctly convex elytral sutural intervals and the tips of the parameres acute and curved ventrally in lateral view. Due to a lack of further material being available of the later species, *B.meridionalis* and *B.minutus* can only be separated by the shape of male genitalia and the lateral margin of the pronotum in *B.meridionalis*, which is more widely explanate than that of *B.minutus*.

#### Chinese name.

脊背厚角金龜

#### Notes.

Li et al. (2008) excluded *B.meridionalis* from the registered Taiwan fauna due to the lack of verified records. During the present study, we examined a male *B.meridionalis* specimen housed in NSMT bearing identical labels as the paratype of the species collected in Heito (now Pingtung) by the late Japanese coleopterist, Yushiro Miwa. We therefore confirm the record of *B.meridionalis* in Taiwan, though it has been lacking in reports of the genus for 90 years. Consequently, the conservation status of *B.meridionalis* in Taiwan is in urgent need of study, along with that of *B.minutus* Li & Masumoto, 2008 and *Bolbotrypesdavidis* (Fairmaire, 1891). These species are restricted to habitats in highly urbanized areas and/or intensively farmed plains of Taiwan.

#### Distribution.

Indonesia (Java); eastern China; Thailand; Vietnam; Taiwan.

#### Remarks.

*Bolbelasmusmeridionalis* has the widest known distribution among congeners in the region in eastern and southeastern Asia. Also, the records from Java for the holotype and paratypes indicated that it is the only member from the Sunda Islands of the genus.

### ﻿ Bolbelasmus minutus

Taxon classificationAnimaliaColeopteraGeotrupidae

Li & Masumoto, 2008

96D9C6B6-38E4-5F68-82FA-FB32CD2C99DC

Bolbelasmus minutus Li & Masumoto, 2008: 482. Original combination (type locality: Heito (presently Pingtung), Taiwan); Nikolajev, Král and Bezdӗk 2016: 33 (catalog, subgenus Kolbeus).

#### Distribution.

Taiwan.

#### Remarks.

*Bolbelasmusminutus* was described from a pair of specimens collected during 1931, and no further records of the species have been recorded. This species occurs sympatrically with *B.meridionalis* and *B.nativus* in the plains of southern Taiwan.

### ﻿ Bolbelasmus nativus nativus

Taxon classificationAnimaliaColeopteraGeotrupidae

Krikken, 1977

34EDE9E6-1640-502A-A9D4-7695608C0F8B

Bolbelasmus nativus Krikken, 1977: 287. Original combination (type locality: Heito (presently Pingtung), Taiwan); Nikolajev, Král and Bezdӗk 2016: 33 (catalog, in nominate subgenus Bolbelasmus).
B. n. ishigakiensis ssp. Masumoto, 1984.
Bolbelasmus ishigakiensis Masumoto, 1984: 73. Original combination (type locality: Ishigaki island, Okinawa, Japan); Král, Löbl and Nikolajev 2006: 83 (catalog, subgenus Kolbeus); Ochi and Masumoto 2005: 244 (as subspecies of B.nativus); Nikolajev, Král and Bezdӗk 2016: 33 (catalog, subgenus Kolbeus).

#### Distribution.

Taiwan (southern areas and Lanyu island); Japan (Iriomote, Ishigaki and Tarama islands, Okinawa Prefecture).

#### Remarks.

*Bolbelasmusnativus* was originally described based on a single male from Taiwan. Ochi and Masumoto (2005) treated the population distributed on a few small islands near Taiwan as a subspecies, *B.nativusishigakiensis*.

### ﻿ Bolbelasmus shibatai

Taxon classificationAnimaliaColeopteraGeotrupidae

Masumoto, 1984

EA0192B9-1149-5636-96F4-87E9239620EA

Bolbelasmus shibatai Masumoto, 1984: 75. Original combination (type locality: Amami Oshima Island, Japan); Nikolajev, Král and Bezdӗk 2016: 33 (catalog).

#### Distribution.

Japan (Amami oshima and Okinawa island).

#### Remarks.

Populations of *B.shibatai* are restricted to a few small islands in the southwestern archipelagos of Japan. Males possess strongly sclerotized parts of the parameres that can be distinguished from the similar species, *B.coreanus*.

### ﻿ Bolbelasmus yutangi

Taxon classificationAnimaliaColeopteraGeotrupidae

Li & Wang sp. nov.

4203E0EB-4F02-5F3D-9356-74590CF2005F

https://zoobank.org/A57879F9-F8A6-4D40-B922-DF3429C8BA4D

Figs 9 , 10 , 15 , 21 , 27 , 37 , 38 , 43 , 50 , 51

#### Type materials.

***Holotype* male.** “Myanmar: Bago Region, Moe Yin Gyi, CCCC, 21.V.2017. leg. Y.-T. Wang.” (glued on label, TARI). ***Paratypes*.** 1♀(TARI). data as the holotype. **5**♂♂ (TARI). “China: Yunnan, Wudian (武甸), 17.IX.2014. leg. Y.-T. Wang”. 1♀ (CCLI) “China: Yunnan, Banggunjianshan (邦棍尖山), 18.IX.2015. leg. Y.-T. Wang”. 6♂♂ (CCLI). “China: Yunnan, Bangdashan (邦達山), 01.IX.2015. leg. Y.-T. Wang”. 1♂ (CCLI). “China: Yunnan, Ruili (瑞麗), 15.IX.2014. leg. Y.-T. Wang”. 1♂ (NSMT). “Doi Saket, Chiang Mai, Thailand, 12-X-1989, Y. MANIT leg”. 1♂ (NSMT). “Doi Mon Unggate, Samoeng Distr., Chiang Mai Prov., Thailand, 18-VII-1989, Y. MANIT leg”.

#### Description.

**Holotype male** (Figs 9, 10). Body length 9.3 mm; width across humeri 5.9 mm. Dorsum moderately shiny. Head, pronotum and scutellum dark brown with elytra reddish brown. ***Head*** (Fig. 15): labrum with anterior margin crenulate, disc transversally rugose. Clypeus trapezoidal, surface densely rugopunctate; protrusion at basal angle moderately developed. Clypeofrontal suture well defined, slightly curved in front of frontal tubercle. Frons with surface moderately punctate, punctures coarse, frontal tubercle vertically located at center of disc, triangular when viewed laterally. Eye prominent, canthus simple, not exceeding eye. ***Thorax***: pronotum (Fig. 21) quadrituberculate, tubercles placed in a line, lateral tubercle greatly reduced in size; anterior face of median tubercles almost perpendicular to plane of pronotum; primary punctures coarse, dense on disc with small impunctate area in front of middle of posterior margin, punctures between lateral margins of pronotum and fovea bigger and denser, scattered coarse punctures distributed along posterior margin with seven punctures in front of scutellum, secondary punctures tiny, evenly scattered on surface of pronotum; frontal and lateral margins beaded, posterior margin beaded only in front of elytral humeri. Scutellum elongate, fine punctures sparsely distributed on surface. ***Elytron*** (Fig. 27): elytral striae shallowly impressed, punctures mostly spaced by 1–3 times diameters of punctures. Intervals slightly convex, including sutural interval, surface with scattered secondary punctures. Male genitalia. Figs 37, 38, 43.

**Figures 29–38.**
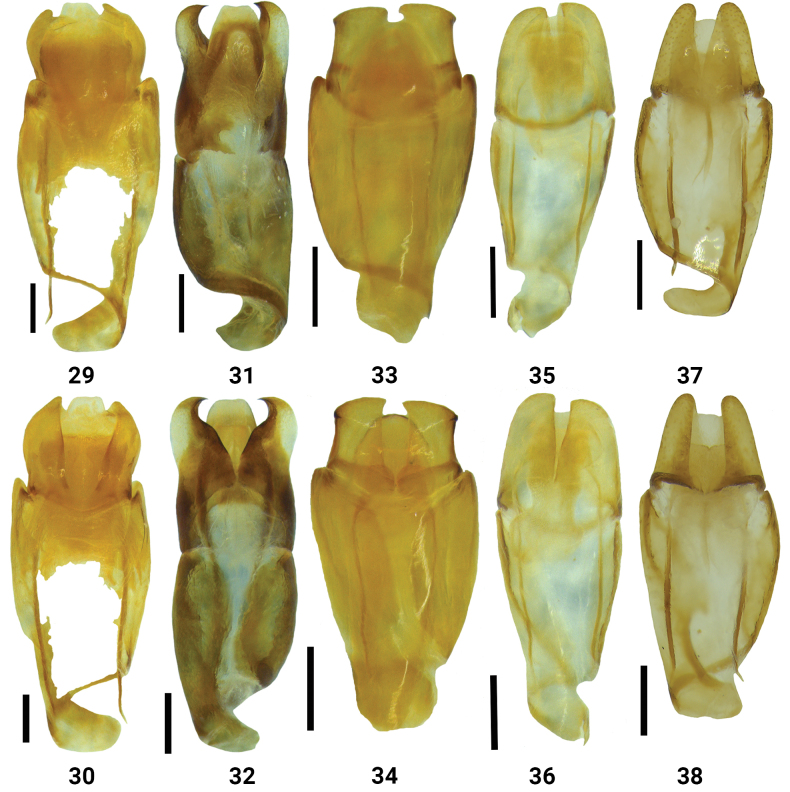
Male genitalia of *Bolbelasmus* spp. (29, 31, 33, 35, 37, dorsal views; 30, 32, 34, 36, 38, ventral views) **29, 30***B.concavisuturalis* sp. nov. **31, 32***B.chifengi* sp. nov. **33, 34***B.meridionalis***35, 36***B.korshunovi***37, 38***B.yutangi* sp. nov. Scale bar: 0.3 mm.

**Figures 39–43.**
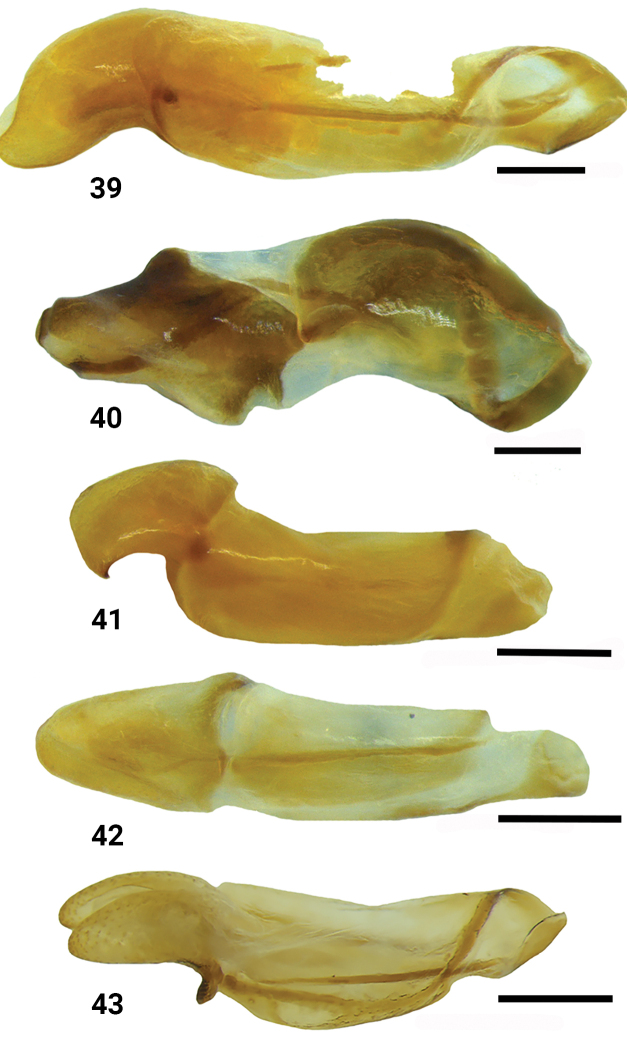
Male genitalia of *Bolbelasmus* spp., lateral views **39***B.concavisuturalis* sp. nov. **40***B.chifengi* sp. nov. **41***B.meridionalis***42***B.korshunovi***43***B.yutangi* sp. nov. Scale bar: 0.2 mm.

**Female** (Figs 50, 51). Body length 8.4–8.7 mm; width across humeri 5.1–5.3 mm. Similar to male with minor differences in the form of strongly wrinkled surface of clypeus, transverse frontal carina trilobed, central lobe more prominent than lateral lobes, punctures on frons and vertex rugose, transverse pronotal carina feebly bilobed, with lobes broadly developed to reduced, bigger punctures on pronotal disc denser than those of males and scutellum with 1 or 2 bigger punctures.

**Figures 44–51.**
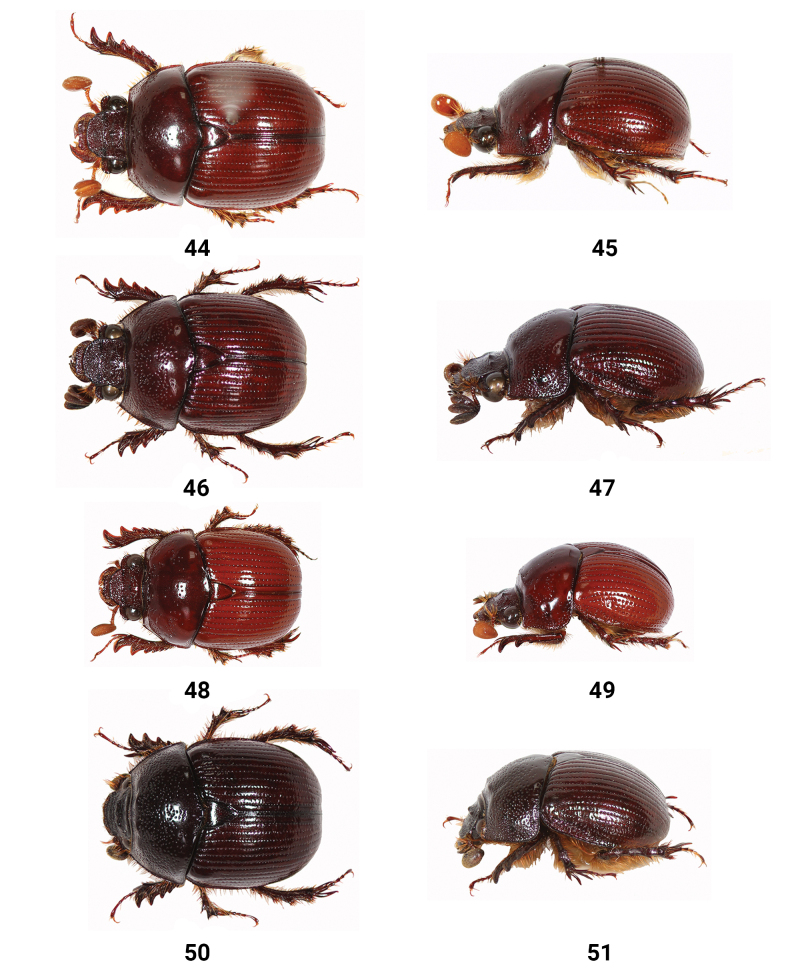
Dorsal and lateral views of female *Bolbelasmus* spp. **44, 45***B.concavisuturalis* sp. nov., paratype **46, 47***B.chifengi* sp. nov., paratype **48, 49***B.meridionalis***50, 51***B.yutangi* sp. nov., paratype.

**Variation in male.** Male paratypes differ from the holotype in the following respects: smaller body size, 6.6 mm in body length and 5.2 mm in width across humeri, frontal tubercle less developed and not in junction of clypeofrontal suture, pronotal tubercle feebly convex, reduced, and number of coarse punctures along pronotal posterior margin variable.

#### Diagnosis.

*Bolbelasmusyutangi* is similar to *B.nativus* in sharing the frontal tubercle location at the center of the frons as well as having smaller parameres. It can be distinguished from the latter by the weakly convex basal angle of the clypeus (distinctly bulging in *B.nativus*), primary punctures densely distributed on the disc (almost impunctate along the middle of disc in *B.nativus*), sutural interval convex, similar to discal intervals (distinctly more convex than discal intervals in *B.nativus*) and parameres with the tips tapered (parameres broader at tips in *B.nativus*).

#### Distribution.

**Figure 52.**
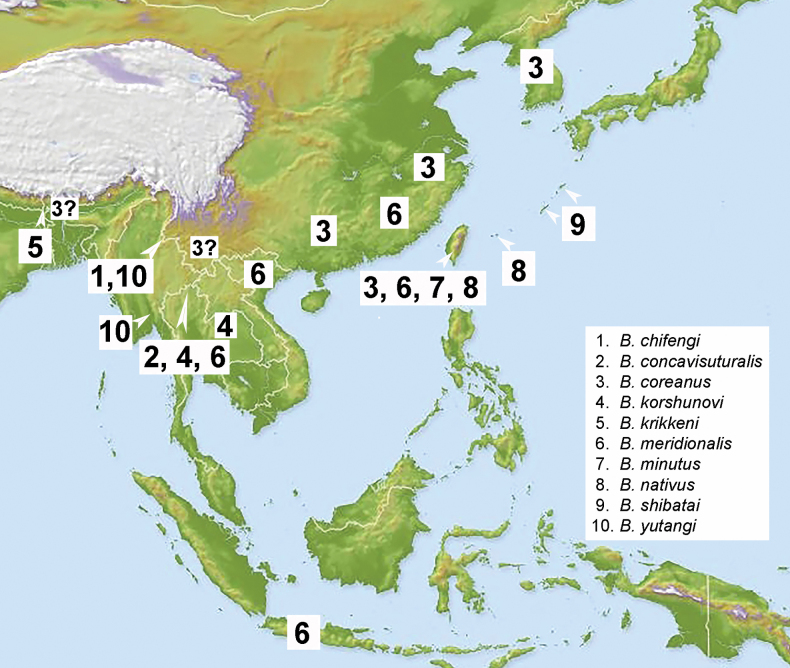
Distribution map of the eastern and southeastern Asian *Bolbelasmus* species. Question marks indicate doubtful distributional records.

Eastern Myanmar, northern Thailand and western Yunnan, China (Fig. 52).

#### Etymology.

*Bolbelasmusyutangi* sp. nov. is named after Mr Yu-tang Wang, a beetle enthusiast of Taiwan, who collected most of the material used in this study.

##### ﻿Species with doubtful locality record

### ﻿ Bolbelasmus orientalis

Taxon classificationAnimaliaColeopteraGeotrupidae

Petrovitz, 1968

4E4E95D6-287F-52E9-B77D-452832EF4044

Bolbelasmus orientalis Petrovitz, 1968: 185. Original combination (type locality: Wladiwostok [Vladivostok], Primorskii Territory, Russia); Krikken 1977: 289 (notes; diagnosis; illustration); Nikolajev, Král and Bezdӗk 2016: 33 (catalog).

#### Distribution.

Described from Vladivostok, Russian Far East.

#### Remarks.

*Bolbelasmusorientalis* was described from one male and one female. Krikken (1977) examined both type specimens and found that the female allotype to be a minor male. Also, he noted that the species has no direct affinity with three congeners, *B.coreanus*, *B.meridionalis* and *B.nativus*, which are geographically close to *B.orientalis* but are allied to the western Palaearctic *B.unicornis*. Bezborodov and Koshkin (2014) doubted the locality label attached to the type specimens because there were no additional records of the species documented in Russia or nearby territories other than that of type specimens. We, therefore, exclude *B.orientalis* from the *Bolbelasmus* fauna in the eastern Palaearctic and the Oriental regions.

## Supplementary Material

XML Treatment for Bolbelasmus

XML Treatment for Bolbelasmus chifengi

XML Treatment for Bolbelasmus concavisuturalis

XML Treatment for Bolbelasmus coreanus

XML Treatment for Bolbelasmus korshunovi

XML Treatment for Bolbelasmus krikkeni

XML Treatment for Bolbelasmus meridionalis

XML Treatment for Bolbelasmus minutus

XML Treatment for Bolbelasmus nativus nativus

XML Treatment for Bolbelasmus shibatai

XML Treatment for Bolbelasmus yutangi

XML Treatment for Bolbelasmus orientalis

## ﻿Additional information

### Data availability

All of the data that support the findings of this study are available in the main text.

## References

[B1] Bezborodov VG Koshkin ES (2014) A review of Bolboceratidae (Coleoptera, Scarabaeoidea) species from the Russian Far East. Entomological Review 93(8): 953–959. 10.1134/S0013873814090127

[B2] Boucomont A ([1911] 1910) Contribution à la classification des Geotrupidae. Annales de la Société entomologique de France. Paris (1911) 79: 333–350. 10.5962/bhl.part.4697

[B3] Boucomont A (1912) Scarabaeidae: Taurocerastinae, Geotrupinae. In: Junk W Schenkling S (Eds) Coleopterorum catalogus, pars 46.W. Junk, Berlin, 1–47.

[B4] Boucomont A Gillet J (1921) Faune entomologique de l’Indo-chine française. Fam. Scarabaeidae Laparosticti. Imprimerie Nouvelle Albert Portail. Saigon 4: 1–76.

[B5] Cartwright OL (1953) Scarabaeid beetles of the genus *Bradycinetulus* and closely related genera in the United States. Proceedings of the United States National Museum 103(3318): 95–120. 10.5479/si.00963801.103-3318.95

[B6] Fairmaire L (1896) Coléoptères de l’Inde boréale, Chine et Malaysie. Notes of the Leyden Museum 18: 81–129.

[B7] Hillert O Arnone M Král D Massa B (2016) The genus *Bolbelasmus* in the western and southern regions of the Mediterranean Basin. Acta Entomologica Musei Nationalis Pragae 56(1): 211–254.

[B8] Kawai S Hori S Kawahara M Inagaki M (2005) Atlas of Japanese Scarabaeoidea, volume 1 Coprophagous group. Roppon-Ashi Entomological Books, Tokyo, 189 pp.

[B9] Kim JI (2000) Scarabaeoidea (I). Economic Insects of Korea, 4. Ins. Koreana Suppl. 11. National Institute of Agricultural Science and Technology, Suwon, 149 pp.

[B10] Kolbe HJ (1886) Beiträge zur Kenntniss der Coleopteren Fauna Koreas. Archiv für Naturgeschichte 52: 139–240. 10.5962/bhl.part.28437

[B11] Král D Löbl I Nikolajev GV (2006) Bolboceratidae. In: Löbl I Smetana A (Eds) Catalogue of Palaearctic Coleoptera, volume 3.Apollo Books, Stenstrup, Denmark, 82–84. 10.1163/9789004260917_004

[B12] Krikken J (1977) The genus *Bolbelasmus* Boucomont in Asia, with notes on species occurring in other regions (Coleoptera: Geotrupidae). Zoölogische Mededeelingen 51: 277–292. https://repository.naturalis.nl/pub/319326

[B13] Li CL Wang CC Masumoto K Ochi T Yang PS (2008) Review of the tribe Bolboceratini s.l. from Taiwan (Coleoptera: Scarabaeoidea: Geotrupidae) with a key to the Eurasian genera. Annals of the Entomological Society of America 101(3): 474–490. 10.1603/0013-8746(2008)101[474:ROTTBS]2.0.CO;2

[B14] Masumoto K (1984) New coprophagous lamellicornia from Japan and Formosa (I). Entomological Review of Japan 39: 73–83. https://archive.org/details/entomologicalreviewofjapan19840039001/page/n75/mode/2up

[B15] Miwa Y (1930) An enumeration of the coprophagid-coleoptera from Formosa, with a table of the geographical distribution. Insecta Matsumurana IV(4): 163–180.

[B16] Miwa Y (1931) A systematic Catalogue of Formosan Coleoptera. Report of the Department of Agriculture of the Government Research Institute 55: 1–359.

[B17] Miwa Y Chûjô M (1939) Scarabaeidae. Catalogus Coleopterorum Japonicorum, Pars 5. Noda-Syobo, Taihoku, 94 pp.

[B18] Nikolajev GV (1979) Eine neue *Bolbelasmus*-Art aus Asien (Coleoptera, Scarabaeidae, Bolboceratinae). Reichenbachia 17: 225–227.

[B19] Nikolajev GV Král D Bezdӗk B (2016) Bolboceratinae. In: Löbl I Löbl D (Eds) Catalogue of Palaearctic Coleoptera, volume 3.Koninklijke Brill NV, Leiden, The Netherlands, 33–36. 10.1163/9789004309142

[B20] Ochi T Masumoto K (2005) Systematic position of *Bolbelasmusishigakiensis* Masumoto (Coleoptera, Scarabaeidae). Elytra 3: 244. https://archive.org/details/elytra20050033001/page/n245/mode/1up

[B21] Paulian R (1945) Faune de l’empire français III coléoptères Scarabéides de l’Indochine. Librairie Larose, Paris, 227 pp.

[B22] Petrovitz R (1968) Bekannte und unbekannte Scarabaeidae (Hybosorinae, Troginae, Orphninae, Dynamopinae, Geotrupinae, Aegialinae). Entomologische Arbeiten aus dem Museum G. Frey 19: 179–187. https://www.zobodat.at/pdf/Entomologische-Arbeiten-Museum-Frey_19_0179-0187.pdf

[B23] Schoolmeesters P (2023) World Scarabaeidae Database. In: Bánki O, Roskov Y, Döring M, Ower G, Vandepitte L, Hobern D, Remsen D, Schalk P, DeWalt RE, Keping M, Miller J, Orrell T, Aalbu R, Abbott J, Adlard R, Adriaenssens EM, Aedo C, Aescht E, Akkari N, et al. (Eds) Catalogue of Life Checklist (Version 2023-02-06).

[B24] Tsukamoto K Inagaki M Kawahara M Mori M (2017) *Geotrupes* and related species of Japan, Handbook Series of Insects 9. Mushi-sha, Tokyo, 116 pp.

[B25] Zinchenko VK (2016) A new species of the genus *Bolbelasmus* (Coleoptera, Bolboceratidae) from Thailand. Evraziatskii Entomologicheskii Zhurnal 15(4): 328–329. https://kmkjournals.com/upload/PDF/EEJ/15/EEJ15_4_06.pdf

